# The Role of Pneumococcal and Human Papillomavirus Vaccination in Preventing Otolaryngological Diseases: Implications for Pediatric Practice

**DOI:** 10.3390/children13070958

**Published:** 2026-07-21

**Authors:** Carla Ungaro, Flavia Oliva, Giuseppe Ricciardiello, Teresa Abate, Marina Tesorone

**Affiliations:** 1 U.O.S.D. Coordination of Clinical Activities in Childhood Obesity, ASL Napoli 1 Centro, 80145 Naples, Italy; 2 Division of Otolaryngology, “A. Cardarelli” Hospital, 80131 Naples, Italy; oliva.flavia@libero.it (F.O.); abateteresa@libero.it (T.A.); 3 Department of Mental Health and Public Medicine, Section of Infectious Diseases, University of Campania Luigi Vanvitelli, 80138 Naples, Italy; ricciardiellogiuseppe@libero.it; 4 U.O.C. Protection of Women, Child and Adolescent’s Health, Local Health Unit, ASL Napoli 1 Centro, 80145 Naples, Italy; marina.tesorone@aslnapoli1centro.it

**Keywords:** pneumococcus, acute otitis media, pneumococcal conjugate vaccines, human papillomavirus, oropharyngeal carcinoma, prophylactic papillomavirus vaccination

## Abstract

**Highlights:**

**What are the main findings?**

- Higher-valency pneumococcal vaccines significantly reduce severe acute otitis media, related complications, and the need for surgical interventions in children.
- Human papillomavirus vaccination effectively prevents oral infection with high-risk viral types in adolescents and young adults.
- Vaccination programs have changed the epidemiology of pediatric otolaryngological infections, highlighting the emergence of non-vaccine pathogens and serotypes.

**What are the implications of the main findings?**

- Achieving and maintaining high vaccination coverage is essential to reduce the burden of preventable otolaryngological diseases in childhood.
- Human papillomavirus vaccination may contribute to the primary prevention of oropharyngeal cancer by reducing oral viral infections.
- Continuous epidemiological surveillance and the development of broader-coverage vaccines are needed to sustain and improve preventive effectiveness.

**Abstract:**

Vaccinations are a cornerstone of primary prevention in pediatric otolaryngology and play a critical role in reducing the burden of pediatric infectious diseases, particularly acute otitis media (AOM) and HPV-related infections. The introduction and widespread use of pneumococcal conjugate vaccines (PCVs) have been associated with substantial changes in the epidemiology of pediatric AOM, particularly through reductions in vac-cine-serotype disease and complicated forms, while contributing to changes in pathogen distribution with an increased role of non-vaccine serotypes and non-pneumococcal bac-teria, requiring continuous epidemiological surveillance. Prophylactic human papillomavirus (HPV) vaccination in adolescents and young adults effectively prevents oral infection with vaccine-type high-risk HPV, a necessary step in the pathogenesis of HPV-related oropharyngeal carcinoma. Clinical and epidemiological studies demonstrate robust antibody responses and significant reductions in oral HPV prevalence among vaccinated individuals. Although direct evidence of a reduction in oropharyngeal cancer incidence is not yet available, vaccination may consequently reduce the future burden of HPV-related oropharyngeal cancers. Long-term surveillance remains essential to confirm this potential benefit. Barriers to optimal vaccine coverage include unequal access, incomplete vaccination uptake, and limited awareness of HPV-related oral disease. In conclusion, vaccinations provide direct and clinically relevant benefits in pediatric otolaryngology by reducing recurrent AOM, severe complications and surgical interventions. HPV vaccination also reduces vaccine-type oral HPV infection and may consequently contribute to reducing the future burden of HPV-related oropharyngeal cancers. Achieving high vaccination coverage, coupled with continuous epidemiological monitoring, is essential to maximize individual and public health benefits and to inform the development of vaccines with broader serotype coverage.

## 1. Introduction

Vaccination represents one of the most effective preventive strategies in pediatric healthcare and has substantially reduced the burden of infectious diseases and their com-plications. In pediatric otolaryngology, vaccine-preventable conditions continue to ac-count for a considerable proportion of outpatient visits, antibiotic prescriptions, hospital-izations, and surgical procedures. Among these conditions, acute otitis media (AOM) re-mains one of the most common childhood infections, while human papillomavirus (HPV) infection is a preventable cause of oral infection and a well-established risk factor for HPV-related oropharyngeal carcinoma later in life.

Otological and upper respiratory tract infections continue to impose a substantial global health burden in children. AOM is among the most common pediatric infections, with peak incidence between 6 and 24 months of age, and is complicated by otorrhea in approximately 10–15% of cases. Recurrent and complicated forms of otitis media are as-sociated with significant morbidity, including hearing impairment, delayed speech and language development, reduced quality of life, and, in selected cases, the need for surgical intervention, such as tympanostomy tube insertion. In addition to their clinical impact, these conditions are responsible for considerable healthcare utilization and costs owing to repeated medical consultations, antibiotic prescriptions, emergency department visits, and hospital admissions. Their burden remains particularly high in low- and middle-income countries, where access to preventive measures and specialist care is often limited [1].

Similarly, HPV infection has become a major public health concern because of its es-tablished association with several malignancies, including oropharyngeal carcinoma. Although these cancers generally develop during adulthood, primary prevention through HPV vaccination is implemented during childhood and adolescence, providing long-term protection against HPV infection and its related diseases [2]. Consequently, pediatric im-munization programs may yield health benefits that extend well beyond childhood.

Despite the availability of safe and effective vaccines, coverage rates for both pneu-mococcal conjugate vaccines (PCVs) and HPV vaccines remain suboptimal in many re-gions worldwide. Vaccine hesitancy, disparities in healthcare access, and differences in national immunization policies continue to limit the full public health benefits of these vaccination programs. Furthermore, the role of vaccination in preventing otolaryngologi-cal diseases is often underrecognized in routine pediatric practice. Pediatricians play a pivotal role in the management of recurrent AOM, counseling families on vaccine uptake, reducing unnecessary antibiotic use, and promoting preventive healthcare. However, evi-dence regarding the impact of pneumococcal vaccination on the incidence and severity of AOM and its complications, as well as the effectiveness of HPV vaccination in preventing oral HPV infection and potentially reducing the future burden of HPV-related oropharyn-geal disease, remains scattered across different medical specialties.

A comprehensive synthesis of the available evidence may facilitate evidence-based counseling and support preventive strategies in pediatric clinical practice. The aim of this narrative review is to summarize current evidence on the effectiveness of pneumococcal vaccination in reducing the incidence, severity, and complications of AOM, as well as the role of HPV vaccination in preventing oral HPV infection and its potential contribution to reducing the future burden of HPV-related oropharyngeal disease. Particular emphasis is placed on the implications of these findings for pediatric clinical practice, vaccine coun-seling, and public health prevention strategies.

## 2. Materials and Methods

This manuscript presents a narrative review of the literature focusing on the role of pneu-mococcal and HPV vaccination in the prevention of otolaryngological diseases in the pe-diatric population. The aim of this review was to provide a clinically oriented synthesis of the current evidence and to summarize the implications of vaccination strategies for pedi-atric practice, rather than to perform a systematic review or meta-analysis.

### 2.1. Literature Search Strategy

A structured literature search was conducted in PubMed and Scopus. Mendeley Reference Manager was used as a reference management tool to organize retrieved records and iden-tify additional relevant publications through citation management. Additional sources in-cluded the Cochrane Library, the Centers for Disease Control and Prevention (CDC), and relevant European public health institutional websites. The search included articles pub-lished from January 2008 to March 2026. The starting date was selected because it corre-sponds to the period following the widespread implementation of pneumococcal conju-gate vaccination programs in many countries and includes contemporary evidence on the impact of PCV7, PCV10, PCV13, and subsequent higher-valency vaccines, and the evolu-tion of HPV vaccination strategies. Only articles published in English were considered el-igible.

The search strategy was developed to identify clinically relevant evidence rather than to perform a systematic review. Database-specific searches were conducted using combina-tions of controlled vocabulary terms (Medical Subject Headings [MeSH] in PubMed where applicable) and free-text keywords adapted to each database. Because this was a narrative review, the same core search concepts were applied across databases with minor syntax modifications. A representative search strategy was as follows: (“pneumococcal conjugate vaccine” OR “pneumococcal vaccination” OR PCV7 OR PCV10 OR PCV13 OR PCV15 OR PCV20) AND (“acute otitis media” OR otitis media OR ENT OR otolaryngology) AND (child* OR pediatric OR paediatric); and (“human papillomavirus vaccine” OR HPV vac-cine OR HPV vaccination) AND (“oral HPV” OR “oropharyngeal cancer” OR “head and neck cancer”) AND (child* OR adolescent* OR pediatric OR paediatric). Equivalent search syntax was adapted for Scopus and the Cochrane Library.

### 2.2. Study Selection

Eligible publications included original research articles, randomized controlled trials, co-hort studies, case-control studies, systematic reviews, meta-analyses, and relevant narra-tive reviews evaluating the impact of pneumococcal and/or HPV vaccination on otolaryn-gological outcomes or related infectious diseases. Studies were excluded if they were not published in English, did not address pediatric populations or pediatric-relevant preven-tive strategies, lacked relevance to otolaryngological or upper respiratory tract outcomes, were conference abstracts without full-text availability, or represented duplicate publica-tions. Study selection was based on title and abstract screening followed by full-text eval-uation of potentially relevant articles. Because this work was designed as a narrative re-view rather than a systematic review, the search process aimed to identify the most clini-cally relevant evidence rather than to achieve exhaustive study retrieval. No formal PRISMA flow diagram was generated. The narrative synthesis included 40 publications considered most relevant to the objectives of this narrative review and representing the core evidence discussed in the manuscript. The included studies were selected according to clinical relevance, methodological robustness, consistency of findings, and applicability to pediatric clinical practice.

### 2.3. Data Extraction and Synthesis

For each included publication, the following information was extracted: study design, population characteristics, vaccine type and formulation, evaluated outcomes, and main findings related to otolaryngological disease prevention. Given the narrative nature of the review, no formal risk-of-bias assessment or quantitative quality scoring was performed. No formal quality scoring system was applied because the objective was to provide a clin-ically oriented narrative synthesis rather than a systematic evidence assessment. Instead, methodological robustness was judged qualitatively according to study design, sample size, appropriateness of outcome measures, duration of follow-up, relevance of the study population, and consistency with findings from other investigations. Evidence was syn-thesized qualitatively, integrating findings from clinical studies, epidemiological analyses, systematic reviews, and public health recommendations. Due to substantial heterogeneity in study populations, vaccine formulations, outcomes, and study designs, a meta-analysis was not performed.

## 3. Results

### 3.1. Effectiveness Data and Ear, Nose, and Throat (ENT) Impact of Pneumococcal Vaccination

Streptococcus pneumoniae (S. pneumoniae) is a widespread bacterium for which humans are the only obligate host and is commonly found in the upper respiratory tract of healthy children and adults. Strains are differentiated into serotypes based on capsular polysaccharides, and more than 100 different serotypes are currently known.

S. pneumoniae is the most common etiological agent of invasive bacterial diseases such as meningitis and sepsis and is also responsible for pneumonia, upper respiratory tract infections, and otitis media. Individuals at highest risk are children under 5 years of age, older adults, and people with compromised immune systems. Sinusitis and otitis media are often preceded by a viral upper respiratory tract infection; otitis media mainly affects young children, whereas bacterial sinusitis can occur in patients of all ages.

While consolidated national surveillance systems exist for invasive pneumococcal diseases (meningitis, sepsis, severe pneumonia), systematic epidemiological surveillance of pneumococcal otitis media is limited or absent in most countries. The main reason is that otitis media is often diagnosed clinically, without microbiological confirmation. Only small prospective longitudinal studies allow serotype characterization from nasopharyn-geal swabs of children with AOM.

Pneumococcal vaccines are purified antigen vaccines directed against capsulated strains of S. pneumoniae. Two types of pneumococcal vaccines are available, polysaccharide and conjugate vaccines, both based on the induction of an immune response against capsular polysaccharides. The former provides less durable protection by stimulating B lymphocytes only, whereas the latter, by also stimulating T lymphocytes, offers greater protection in terms of both duration and effectiveness.

**Figure 1:**
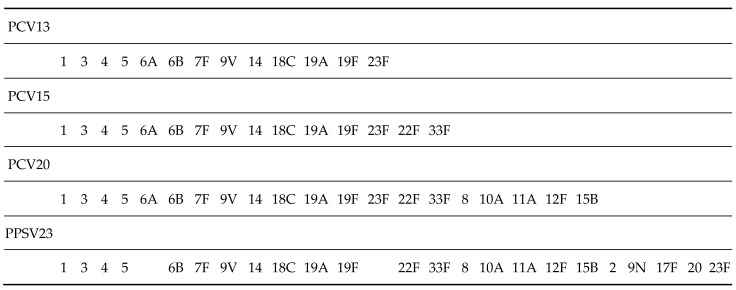
Serotype coverage of pneumococcal vaccines.

The 23-valent pneumococcal polysaccharide vaccine (PPSV23) contains antigens derived from the 23 most virulent pneumococcal serotypes (Figure 1) and is licensed from 2 years of age. Although it protects against a greater number of serotypes, the polysaccharide vaccine is not conjugated, does not induce immunological memory, and is less effective in children under 2 years of age. It is mainly recommended for adults aged ≥65 years, individuals aged ≥2 years with chronic conditions (e.g., cardiovascular diseases, diabetes, chronic obstructive pulmonary disease), and immunocompromised patients, often in sequence with a conjugate vaccine to broaden and strengthen protection. The immune response tends to wane over time; therefore, in individuals with conditions that increase the risk of severe pneumococcal infection, it may be repeated once after 5 years.

PCVs have progressively expanded serotype coverage over the years, evolving from the initial 7- and 10-valent formulations (PCV7 and PCV10) to more recent higher-valence vaccines such as PCV13, PCV15, and PCV20. All can be administered starting from 6 weeks of age (Figure 1). 

Older studies evaluated the effectiveness of lower-valence PCV vaccination on the incidence of otitis media. A systematic review of 18 studies reported that, following the introduction of the 7-valent PCV, medical visits for AOM decreased by an average of 19% in observational studies. However, reductions in otitis-related visits cannot be attributed solely to vaccination, as downward trends were already present before vaccine introduc-tion and were also influenced by multiple factors, including more stringent diagnostic cri-teria, reduced immediate antibiotic prescribing, decreased exposure to passive smoking, and a possible impact of influenza vaccination [3].

Most recent studies have primarily focused on the impact of PCV10 and PCV13 on the incidence, serotype distribution, and overall burden of invasive and non-invasive pneumococcal diseases. Pneumococcal vaccination has led to a marked reduction in invasive pneumococcal infections; however, its impact on AOM remains less consistent, with heterogeneous findings in the literature.

Over the past two decades, the introduction and widespread implementation of pneumococcal vaccines have substantially modified the epidemiology of pediatric AOM, particularly through a reduction in severe and complicated forms. At the same time, changes in pathogen distribution have been observed, including an increased relative contribution of non-pneumococcal bacteria and non-vaccine pneumococcal serotypes, underscoring the need for continuous epidemiological surveillance [4].

More recent observational evidence from studies evaluating PCV10 and PCV13 has reported reductions in outpatient visits for AOM following vaccine introduction. Com-pared with pre-vaccine periods, reductions ranged from 47% to 51% after PCV13 imple-mentation and from 34% to 43% after PCV10 implementation among children younger than 2 years [5].

Further evidence supporting the impact of PCVs on AOM derives from several large-scale studies. A combined randomized and observational analysis by Dagan et al. demonstrated that both the 7-valent and 13-valent PCVs were associated with a significant reduction in the incidence of vaccine-serotype AOM in children, confirming strong sero-type-specific protection [6]. Similarly, a population-based study conducted in Israel by Marom et al. reported a significant reduction in the burden of AOM following the intro-duction of PCVs at the population level, highlighting both direct and indirect (herd) effects [7]. In addition, a Cochrane systematic review by de Sévaux et al. confirmed that PCVs reduce the overall incidence of AOM and related healthcare utilization, although the magnitude of the effect varies according to circulating serotypes and population characteristics [8].

A prospective 18-year longitudinal study (2006–2023) including 1,537 children aged 6–36 months demonstrated that the introduction of PCV7 and subsequently PCV13 was associated with a marked reduction in pneumococcal AOM, particularly in complicated cases. This reduction was driven mainly by a substantial decline in vaccine-included serotypes, especially serotype 19A, which has been historically associated with severe disease and antibiotic resistance [9].

Supporting these findings, a recent meta-analysis showed that PCVs reduce the inci-dence of severe AOM and tympanic membrane perforation and are also associated with a reduced need for surgical intervention for recurrent otitis media, with a 22.2% reduction in tympanostomy tube placement [10].

Although PCV13 has contributed to reducing circulation of several resistant sero-types, pneumococcus remains clinically relevant due to the persistence of non-vaccine serotypes and ongoing antimicrobial resistance. This highlights the importance of contin-uous surveillance and appropriate antibiotic stewardship.

A prospective longitudinal study conducted between 2021 and 2023 in children aged 6–36 months vaccinated with PCV13 evaluated the potential impact of next-generation higher-valent vaccines (PCV15 and PCV20) on nasopharyngeal colonization and AOM prior to their introduction in the United States. Most pneumococcal serotypes identified were not included in PCV13, PCV15, or PCV20, with a predominance of serotype 35B in both colonization and AOM, and frequent detection of other non-vaccine serotypes (23A, 23B, 35D, 35F, 15C). Consequently, the additional coverage provided by PCV15 appeared minimal (0–2%) and only moderate for PCV20 (10–21%), suggesting that the real-world impact of these vaccines on AOM prevention may be limited [11].

Widespread use of PCV13 has also been associated with a shift in the microbiological profile of AOM, with increased relative involvement of Haemophilus influenzae, Moraxella catarrhalis, and Streptococcus pyogenes, the latter being increasingly recognized in complicated AOM with otorrhea [11,12].

Continuous serotype surveillance remains essential to guide vaccine policy and up-date vaccine formulations.

A novel vaccine approved in the United States by the Food and Drug Administration (FDA) for adults aged ≥18 years is PCV21, which covers additional serotypes not included in PCV20 or PCV15 and is primarily intended to reduce invasive disease in older adults, with limited relevance for ENT infections [13].

Future higher-valent formulations, such as PCV31, may provide broader protection and could potentially prevent a larger proportion of invasive pneumococcal disease as well as a subset of respiratory tract infections, including in pediatric populations [13].

### 3.2. Effectiveness Data and ENT Impact of Human Papillomavirus Vaccination (HPV Vaccine)

Human papillomavirus (HPV) belongs to a group of double-stranded DNA viruses that can infect stratified squamous epithelium of the skin and mucosae. More than 230 HPV types have been classified to date. Over 40 types of the Alphapapillomavirus genus infect the anogenital tract and the oral cavity. Based on oncogenic potential, alpha-HPVs are classified as low-risk (LR-HPV) and high-risk (HR-HPV).

The HR group includes 12 types considered carcinogenic, with HPV16 being the most oncogenic. Other types are classified as probably or possibly carcinogenic.

Low-risk types, such as HPV6 and HPV11, can cause benign anogenital warts, low-grade anogenital dysplasia, oral papillomas, and recurrent respiratory papillomatosis.

Persistent infections with high-risk HPV types such as HPV16 may lead to dysplasia and anogenital cancers (cervix, vagina, vulva, penis, anus) as well as oropharyngeal car-cinoma. Among high-risk HPV types, HPV16 remains the most oncogenic, but HPV18 also plays a relevant role in HPV-related malignancies, particularly in epithelial cancers of mucosal origin. Although HPV18 is less frequently detected in oropharyngeal carcinoma compared with HPV16, it contributes significantly to the overall burden of HPV-related cancers [14,15].

A retrospective multicenter study of 1154 adult patients diagnosed with oropharyn-geal squamous cell carcinoma between 2016 and 2020 in four major Canadian oncology centers showed that approximately 80% of oropharyngeal tumors were HPV-related. Most patients were male (85%), and HPV-positive tumors were more frequent in men, younger individuals, and non-smokers. Moreover, these tumors were more often located in the tonsils or the base of the tongue and diagnosed at earlier stages [16].

**Figure 2:**
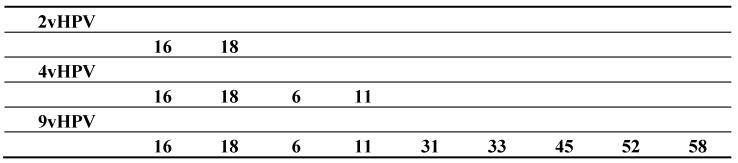
HPV types included in currently used vaccines.

Unlike cervical cancer prevention programs, no screening strategy exists for oropha-ryngeal HPV-related disease. Consequently, primary prevention through vaccination re-mains the most important public health intervention [17]. The vaccine used is a subunit vaccine composed of virus-like particles (VLPs) of the viral capsid, produced in the labor-atory to mimic the structure of the native virus but lacking viral DNA and therefore non-infectious. The currently available nine-valent HPV vaccine (9vHPV) contains virus-like particles (VLPs) derived from HPV types 6, 11, 16, 18, 31, 33, 45, 52, and 58 and has replaced the earlier bivalent HPV vaccine (2vHPV) and quadrivalent HPV vaccine (4vHPV) (Figure 2).

The vaccine is most effective when administered before the onset of sexual activity, underscoring the importance of early adolescent immunization programs. For this reason, international health authorities recommend routine HPV vaccination at 11–12 years of age, with the option to start as early as 9 years, and emphasize the importance of strong, routine recommendations from healthcare providers. The standard vaccination schedule includes two or three doses depending on age at initiation. In several countries, including the United Kingdom, a single-dose HPV vaccination schedule has been authorized and implemented. This approach is supported by evidence from international studies showing that a single dose provides robust protection against the most oncogenic HPV types (nota-bly 16 and 18), with antibody responses and infection prevention comparable to those achieved with a two-dose schedule in adolescents [18]. The Italian National Immunization Prevention Plan has maintained a more cautious approach, retaining the two- or three-dose schedule pending further evidence on long-term protection after a single dose, although reduced-dose schedules may be particularly relevant in settings aiming to im-prove global vaccination coverage.

HPV vaccination is prophylactic and does not have a therapeutic effect on estab-lished infections; however, it prevents new infections with vaccine-covered HPV types. In individuals already HPV-positive at baseline, the additional benefit of vaccination re-mains uncertain [19]. Therefore, its effectiveness is highly dependent on timely admin-istration and high coverage in pre-adolescent populations.

Evidence from clinical and epidemiological studies indicates that prophylactic HPV vaccination in adolescents and young adults reduces oral HPV infection in vaccinated in-dividuals. This is particularly relevant, as oral HPV infection represents a necessary step in the pathogenesis of oropharyngeal carcinoma. However, current evidence is mainly based on surrogate endpoints, such as oral HPV prevalence and immunological respons-es, rather than direct cancer prevention outcomes. Vaccinated individuals develop detect-able anti-HPV16/18 antibodies in oral gargle samples for 18–30 months post-vaccination, although with a gradual decline over time. A large randomized trial conducted in Costa Rica including 7,466 women reported a vaccine efficacy of 93.3% against oral HPV16/18 infection and 91.6% against HPV16 specifically [20]. In addition, a meta-analysis demon-strated that vaccination reduces the risk of oral infection with vaccine-type HPV by ap-proximately 46% and the risk of oral HPV16 infection by approximately 80% [21].

Unlike anogenital neoplasms, persistent oncogenic HPV infections do not typically give rise to well-defined precursor lesions for oropharyngeal carcinoma. Although HPV vaccination is highly effective in preventing oral infection with vaccine-type HPV, direct evidence demonstrating a reduction in oropharyngeal cancer incidence is still lacking. Nevertheless, given the established causal role of HPV, it is biologically plausible that prevention of infection will translate into a future reduction in HPV-related cancers. 

Accordingly, achieving high vaccination coverage in children and adolescents is es-sential to mitigate the expected future burden of HPV-associated oropharyngeal carcino-ma. Several practical and logistical barriers persist, including incomplete vaccination cov-erage, unequal access, non–gender-neutral vaccination policies in some settings, and lim-ited awareness of the association between HPV and oropharyngeal cancer. Long-term studies and surveillance systems are therefore needed to determine whether reductions in oral HPV infection will translate into decreased cancer incidence over time.

Ongoing research is focused on the development of therapeutic HPV vaccines, de-signed not to prevent infection but to eliminate established infection and induce regression of precancerous lesions. Although several therapeutic vaccine candidates have demonstrated immunogenicity and partial efficacy in promoting viral clearance and lesion regression, none has yet achieved sufficient efficacy for regulatory approval [22].

## 4. Discussion

This narrative review highlights the evolving role of PCVs and HPV vaccination in the prevention of otolaryngological diseases in pediatric populations. Overall, vaccination strategies have substantially modified the epidemiology of AOM and reduced the burden of severe and complicated infections, while also providing long-term preventive potential against HPV-related diseases.

In pneumococcal disease, the greatest impact of conjugate vaccines has been observed in severe and complicated forms of AOM rather than in overall incidence. This reflects the multifactorial aetiology of AOM, in which viral pathogens and non-vaccine bacterial species play an increasingly important role following widespread vaccine implementation. Serotype replacement remains a relevant phenomenon and underscores the need for continuous epidemiological surveillance and periodic updates of vaccine formulations. From a clinical perspective, these shifts in pathogen distribution have implications for everyday practice. Pediatricians and otolaryngologists should be aware of the increasing involvement of non-typeable Haemophilus influenzae and Moraxella catarrhalis, particularly in recurrent or complicated cases, which may require adjustments in diagnostic and therapeutic approaches.

Regarding HPV, the primary clinical benefit in pediatric populations lies in preven-tion prior to viral exposure. The highest effectiveness is achieved when immunization is administered during early adolescence, in line with international recommendations. Therefore, pediatricians play a key role in ensuring timely vaccination, addressing vaccine hesitancy, and strengthening routine recommendations during healthcare visits. Although HPV vaccination has demonstrated efficacy in reducing oral HPV infection, evidence of a direct effect on oropharyngeal cancer incidence remains lacking, mainly due to the long latency period of HPV-related carcinogenesis. Nevertheless, the strong biological plausibility supports its role in cancer prevention strategies, which are currently based on reducing infection as a surrogate endpoint. A limitation in interpreting HPV-related evidence is the reliance on intermediate outcomes, such as oral HPV prevalence, rather than long-term oncological endpoints. This distinction is important in clinical counselling, as it clarifies that the benefit of vaccination is preventive at the infectious level rather than immediately measurable in cancer risk reduction.

This narrative review has several strengths. It integrates current evidence on pneu-mococcal and HPV vaccination within a single clinically oriented overview focused on pediatric ENT practice. By combining evidence from infectious diseases, otolaryngology, and preventive medicine, it provides pediatricians and otolaryngologists with a practical framework for vaccine counselling and disease prevention.

However, some limitations should be acknowledged. As a narrative review, this study did not follow a systematic review methodology and no formal risk-of-bias assess-ment was performed. Consequently, selection bias cannot be excluded. Furthermore, evi-dence supporting HPV vaccination for the prevention of oropharyngeal cancer is currently based mainly on surrogate outcomes, such as reductions in oral HPV infection, rather than direct evidence of reduced cancer incidence, owing to the long latency of HPV-associated carcinogenesis.

Future research should prioritize long-term population-based studies evaluating the impact of HPV vaccination on the incidence of HPV-related oropharyngeal cancer, as well as real-world effectiveness studies assessing higher-valent pneumococcal conjugate vac-cines in reducing acute otitis media burden and monitoring serotype replacement. Con-tinued epidemiological surveillance will remain essential to guide future vaccine policy and optimize prevention strategies.

Overall, the integration of pneumococcal and HPV vaccination into pediatric preven-tive care underscores the central role of immunization in reducing both infectious and long-term oncological disease burden.

## 5. Conclusions

In summary, vaccination remains a cornerstone of pediatric preventive care and an essential strategy for reducing the burden of ENT diseases. Pneumococcal conjugate vac-cines have been associated with significant reductions in pneumococcal and complicated acute otitis media, although they do not prevent all episodes because of the multifactorial etiology of the disease and the emergence of non-vaccine serotypes and other bacterial pathogens. Healthcare professionals should therefore continue to recommend age-appropriate pneumococcal vaccination while counselling families about its expected benefits and limitations.

Similarly, HPV vaccination should be routinely recommended before exposure to the virus, as it effectively prevents new infections with vaccine-type HPV but does not elimi-nate established infection or treat existing HPV-related lesions. Early vaccination remains the most effective strategy for reducing the future burden of HPV-associated oropharyngeal cancer.

Finally, continuous epidemiological surveillance is essential to monitor changes in pneumococcal serotype distribution, pathogen replacement, and the long-term effective-ness of HPV vaccination. These data will be fundamental for guiding future vaccine de-velopment, informing public health policies, and optimizing immunization strategies.

## Data Availability

No new data were created or analyzed in this study. Data sharing is not applicable to this article.

## Abbreviations

The following abbreviations are used in this manuscript:

AOM	acute otitis media
CDC	Centers for Disease Control and Prevention
ENT	Ear, nose, and throat
FDA	Food and Drug Administration
HR-HPV	High-risk human papillomavirus
LR-HPV	Low-risk human papillomavirus
PCVs	Pneumococcal conjugate vaccines
PCV13	13-valent pneumococcal conjugate vaccine
PCV15	15-valent pneumococcal conjugate vaccine
PCV20	20-valent pneumococcal conjugate vaccine
PPSV23	23-valent pneumococcal polysaccharide vaccine
*S. pneumoniae*	*Streptococcus pneumoniae*
VLP	Virus-like particle
2vHPV	Bivalent HPV vaccine
4vHPV	Quadrivalent HPV vaccine
9vHPV	Nine-valent HPV vaccine
